# Bioaccumulation and biomagnification of heavy metals in marine micro-predators

**DOI:** 10.1038/s42003-023-05539-x

**Published:** 2023-11-27

**Authors:** Roberto Danovaro, Adele Cocozza di Montanara, Cinzia Corinaldesi, Antonio Dell’Anno, Silvia Illuminati, Trevor J. Willis, Cristina Gambi

**Affiliations:** 1 Dipartimento di Scienze della Vita e dell’Ambiente, Università Politecnica delle Marche, Via Brecce Bianche, 60131 Ancona, Italy; 2 Nature Biodiversity Future Centre, Palermo, Italy; 3 Dipartimento di Scienze e Tecnologie, Università degli Studi di Napoli Parthenope, Centro Direzionale, 80143 Napoli, Italy; 4 Dipartimento di Scienze e Ingegneria della Materia, dell’Ambiente ed Urbanistica, Università Politecnica delle Marche, Via Brecce Bianche, 60131 Ancona, Italy; 5 Department of Integrative Marine Ecology, Stazione Zoologica Anton Dohrn, Fano Marine Center, 61032 Fano, Italy

**Keywords:** Biodiversity, Ecological modelling

## Abstract

Nematodes represent >3/5 of the abundance of the world’s metazoans and usually account for nearly 90% of the total benthic fauna, playing a key ecological role in the benthic ecosystem functioning on a global scale. These small metazoans include a relevant number of microscopic predators and, in turn, are the most abundant preys of macro-megafauna and fish juveniles thus playing a key role in marine food webs. Here, using two independent approaches, we test the bioaccumulation in marine nematodes of several heavy metals present in contaminated sediments. We report here that nematodes, despite their short life cycle and small size, bioaccumulate significantly heavy metals. Bioaccumulation increases from deposit feeders and microalgal grazers to predators of microbes and other tiny metazoans. These results suggest that nematodes also contribute to their biomagnification along the food webs and can contribute to increase the transfer of contaminants from the sediments to larger organisms.

## Abstract (web summary)

Nematodes bioaccumulate contaminants and promote the channeling of heavy metals to higher trophic levels of the food chain. Predators of metazoans display the highest heavy metal concentrations, followed by predators of microbes and microalgal feeders.

## Introduction

Marine nematodes represent 2/3 of the metazoans on Earth. These small-sized organisms (<0.5 mm) are ubiquitous in all marine sediments from shallow waters down to hadal depths. Due to their high abundance, limited mobility, and short life span (typically ranging from 13 to 60 days), nematodes significantly contribute to the biomass production in all marine ecosystems and dominate biomass production at abyssal depths^1,2^. Nematodes include a large variety of feeding guilds (trophic groups), spanning from deposit feeders (that ingest organic detritus from the sediments) and microalgal grazers to predators of microbes (feeding on bacteria and protozoans) and predators of other nematodes and tiny metazoans^3^. In turn, nematodes are the preferred prey of larger organisms and bentho-nekton juveniles^4–8^. Thus, nematodes represent a crucial link between the benthic microbial food web and the higher trophic levels.

Marine sediments are a major reservoir of several pollutants, including heavy metals, as they adsorb on suspended particles, which are then transferred by sedimentation processes on the seafloor^9^. Marine sediments also play a key role in the heavy metals diagenesis, and depending on the environmental conditions, can make xenobiotics more or less bioavailable to marine organisms^10^. Heavy metals bound to the sediment particles can be ingested by deposit-feeding organisms or can be absorbed/uptake directly by microbes and/or microalgae^11–13^. Heavy metals bioaccumulation (i.e., the accumulation of contaminants in the organisms’ tissues during their life^14^) and biomagnification (i.e., the progressive increase of contaminant concentrations in the higher trophic levels^14^) can have severe biological consequences^12,13,15,16^.

Nematodes, due to their direct development and lack of planktonic larvae^6,17^, are permanently exposed to the contaminants present in the sediments and show a different degree of sensitivity to chemical pollution. When nematodes are preyed by fish juveniles and other predators, they can contribute to the transfer of xenobiotics to higher trophic levels. However, the nematode bioaccumulation and biomagnification of contaminants have been so far completely neglected, likely due to their short life span and tiny body size (individual biomass in the order of 0.1 µgC).

To investigate the bioaccumulation and biomagnification potential in marine nematodes we assessed, using two independent methodologies, the content of seven heavy metals (i.e., As, Cd, Cr, Cu, Mn, Ni, and Zn), some of which classified as priority pollutants in marine ecosystems^18,19^, in their tissues. In addition, we explored the relationships between nematode body size and feeding type with their heavy metals’ bioaccumulation.

## Results

### Heavy metals concentration in marine sediments

All selected heavy metals, except for Ni, showed higher concentrations in chronically contaminated sediments of the Bagnoli-Coroglio Bay (hereafter “contaminated sediments”) than in the sediments of the Gabicce Mare in the Adriatic Sea (hereafter “control sediments”) (up to 8-times higher for Zn and As, respectively, Supplementary Table 1). Heavy metal concentrations decreased as follows: Mn > Zn > As > Cr > Cu > Ni > Cd in the Bagnoli-Coroglio Bay, whereas the pattern Mn>Zn>Cr>Ni>Cu>As>Cd was observed in the sediments of the Gabicce Mare.

### Nematode assemblages in contaminated *vs*. control sediments

Nematode abundances ranged from 0.65 × 10^6^ ind m^−2^ (St21) to 3.9 × 10^6^ ind m^−2^ (St127) in contaminated sediments, while they ranged from 1.89 to 2.01 × 10^6^ ind m^−2^ in the control sediments. Nematode assemblages in the contaminated sediments were dominated by microalgal grazers (2A) whose contribution to the total abundance ranged from 38 to 58% (St44 and St127, respectively), followed by deposit feeders (1B) (9–36%, St21, and St19, respectively), predators of microbes (1A) (7–33%, St99 and St44, respectively) and predators of metazoans (2B) (10% and 24%, St99/St127 and St21, respectively). In the control sediments, microalgal grazers represented the dominant group (50%), followed by deposit feeders (25%), predators of metazoans (19%) and predators of microbes (6%), respectively. The individual biomass of nematodes ranged from 0.03 to 0.41 µgC ind^−1^ (Supplementary Fig. 1a) and all trophic groups, except the predators of microbes, typically showed higher values in the contaminated sediments than in control. In contaminated sediments deposit feeders, microalgal grazers and predators of metazoans displayed on average higher individual biomass when compared to predators of microbes while in control sediments this latter group showed higher individual biomass than all other feeding types (Supplementary Fig. 1b). The total nematodes biomass ranged from 60.5 mgC m^−2^ (St21) to 378.6 mgC m^−2^ (St44) in the contaminated sediments, while values ranged from 120.0 to 127.6 mgC m^−2^ in the control site.

### Bioaccumulation in marine nematodes

**Fig. 1:**
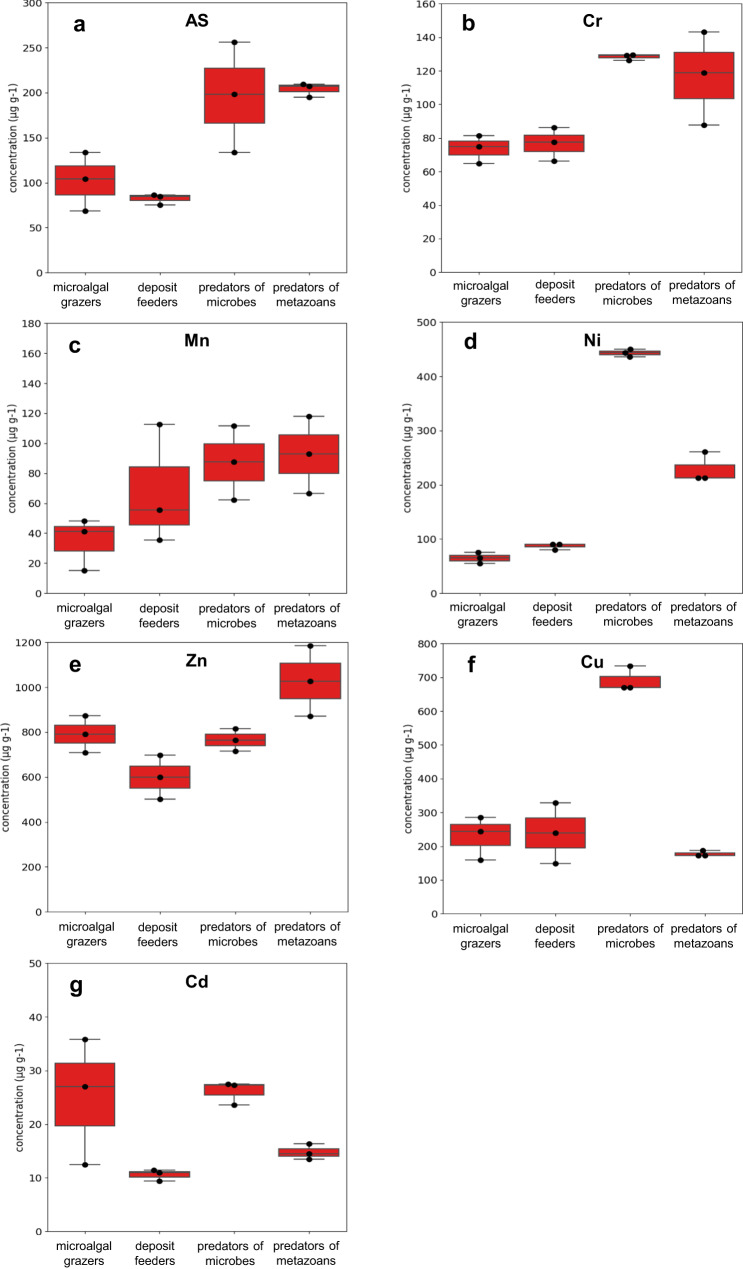
Heavy metal concentration obtained with the atomic absorption spectrophotometer analysis in the four trophic groups. Heavy metals are: **a** As, **b** Cr, **c** Mn, **d** Ni, **e** Zn, **f** Cu and **g** Cd in the four nematode feeding types in the contaminated sediments of Bagnoli-Coroglio Bay. Reported are: microalgal grazers (2A), deposit feeders (1B), predators of microbes (1A) and predators of metazoans (2B). Data are represented as mean ± standard deviation (*n* = 3).

**Fig. 2:**
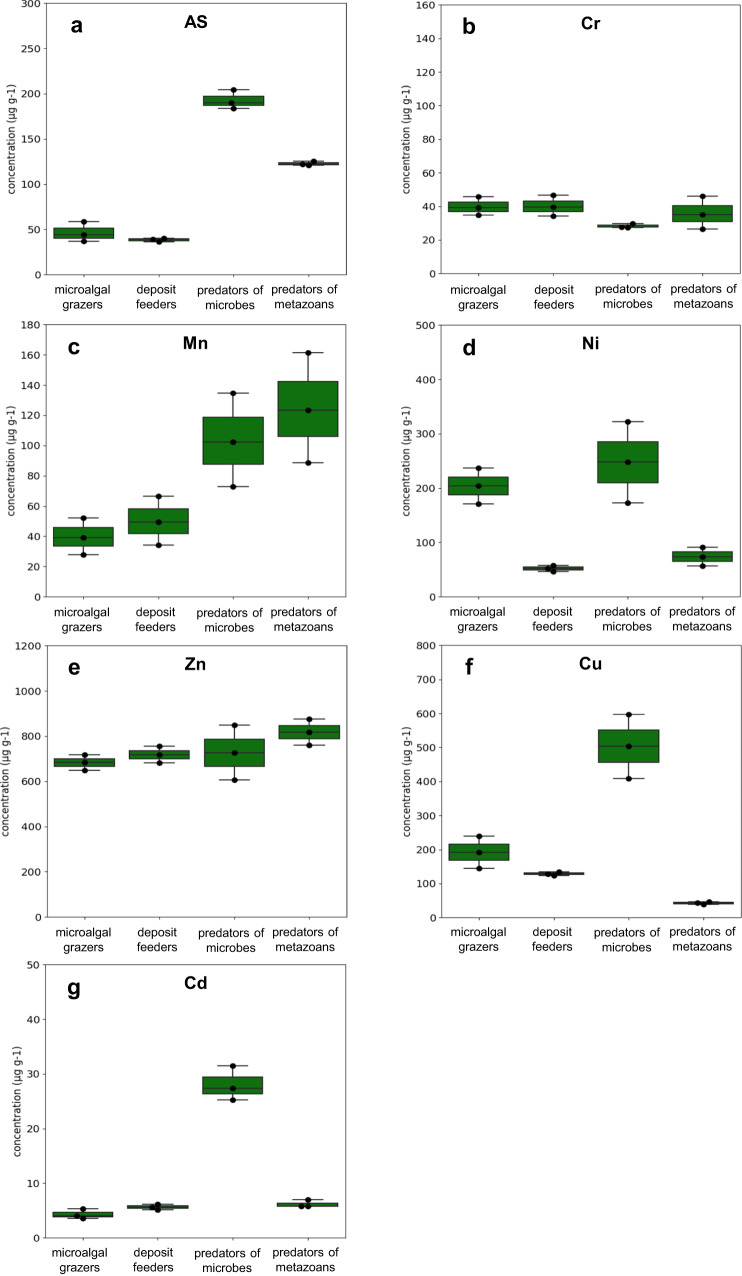
Heavy metal concentration obtained with the atomic absorption spectrophotometer analysis in the four trophic groups. Heavy metals are: **a** As, **b** Cr, **c** Mn, **d** Ni, **e** Zn, **f** Cu and **g** Cd in the four nematode feeding types in Gabicce Mare control site. Reported are: microalgal grazers (2A), deposit feeders (1B), predators of microbes (1A) and predators of metazoans (2B). Data are represented as mean ± standard deviation (*n* = 3).

The results of the atomic absorption spectrophotometer analysis of As, Cd, Cr, Cu, Mn, Ni and Zn concentrations in the four nematode feeding types present in contaminated sediments are reported in Fig. 1a–g. The concentrations of the same heavy metals accumulated in nematodes inhabiting the control site are reported in Fig. 2a–g.

We used the Log-Gamma model (GLM) on the content of heavy metals per feeding type, using the microalgal grazers (herbivores) as baseline of the food web, for testing differences in heavy metal bioaccumulations among feeding types and sediment contamination levels. The output of the GLM indicated that As, Cr, Mn and Ni bioaccumulation in predators of microbes (1A) and metazoans (2B) was significantly higher than in deposit feeders (1B) and microalgal grazers (2A) (2–4 times higher; Table 1). Predators of metazoans (2B) showed also a significantly higher bioaccumulation of Zn, and predators of microbes (1A) showed a significantly higher bioaccumulation of Cu (Table 1). Overall, the content of As, Cd, Cr in nematodes of contaminated sediments was significantly higher than that of nematodes in control sediments (with the only exception of Ni). Finally, Cu, Mn and Zn concentrations did not show significant differences in nematodes from the contaminated and control sediments (Table 1).

**Table 1:** GLM (Log-Gamma Model) estimates of differences in heavy metal concentrations among nematode feeding types and areas (control site of Gabicce Mare vs contaminated sediments of Bagnoli-Coroglio Bay), expressed as odds ratios.

	Contrast	Effect ratio (relative to microalgal grazers)	Lower 95% confidence limit for ratio	Upper 95% confidence limit for ratio	*t*-value	*P*
As	Deposit feeders	0.80	0.60	1.08	−1.44	0.1682
	Predators of microbes	1.92	1.43	2.57	4.34	0.0005
	Predators of metazoans	2.00	1.49	2.68	4.61	0.0003
	Area (control : contaminated sediments)	0.45	0.34	0.61	−5.25	<0.0001
Cr	Deposit feeders	1.04	0.81	1.34	0.30	0.7647
	Predators of microbes	1.74	1.35	2.25	4.24	0.0006
	Predators of metazoans	1.58	1.23	2.05	3.51	0.0029
	Area (control : contaminated sediments)	0.54	0.42	0.70	−4.67	0.0003
Mn	Deposit feeders	1.95	1.07	3.56	2.19	0.0440
	Predators of microbes	2.50	1.37	4.56	2.99	0.0085
	Predators of metazoans	2.66	1.46	4.85	3.19	0.0057
	Area (control : contaminated sediments)	1.14	0.63	2.09	0.44	0.6671
Ni	Deposit feeders	1.34	1.02	1.75	2.12	0.0495
	Predators of microbes	6.82	5.21	8.92	14.01	<0.0001
	Predators of metazoans	3.52	2.69	4.61	9.19	<0.0001
	Area (control : contaminated sediments)	3.14	2.40	4.10	8.34	<0.0001
Zn	Deposit feeders	0.76	0.63	0.91	−2.99	0.0086
	Predators of microbes	0.97	0.81	1.16	−0.35	0.7273
	Predators of metazoans	1.30	1.08	1.56	2.83	0.0121
	Area (control : contaminated sediments)	0.86	0.72	1.04	−1.57	0.1351
Cu	Deposit feeders	1.04	0.75	1.44	0.23	0.8223
	Predators of microbes	3.01	2.17	4.16	6.65	<0.0001
	Predators of metazoans	0.77	0.56	1.07	−1.56	0.1379
	Area (control : contaminated sediments)	0.84	0.60	1.16	−1.08	0.2962
Cd	Deposit feeders	0.42	0.31	0.58	−5.24	<0.0001
	Predators of microbes	1.04	0.75	1.44	0.24	0.8132
	Predators of metazoans	0.59	0.43	0.81	−3.21	0.0054
	Area (control : contaminated sediments)	0.17	0.12	0.24	−10.72	<0.0001

95% confidence limits are asymmetric around the ratio estimate because error estimates are calculated on the log scale and are, therefore, multiplicative on the arithmetic scale.

**Fig. 3:**
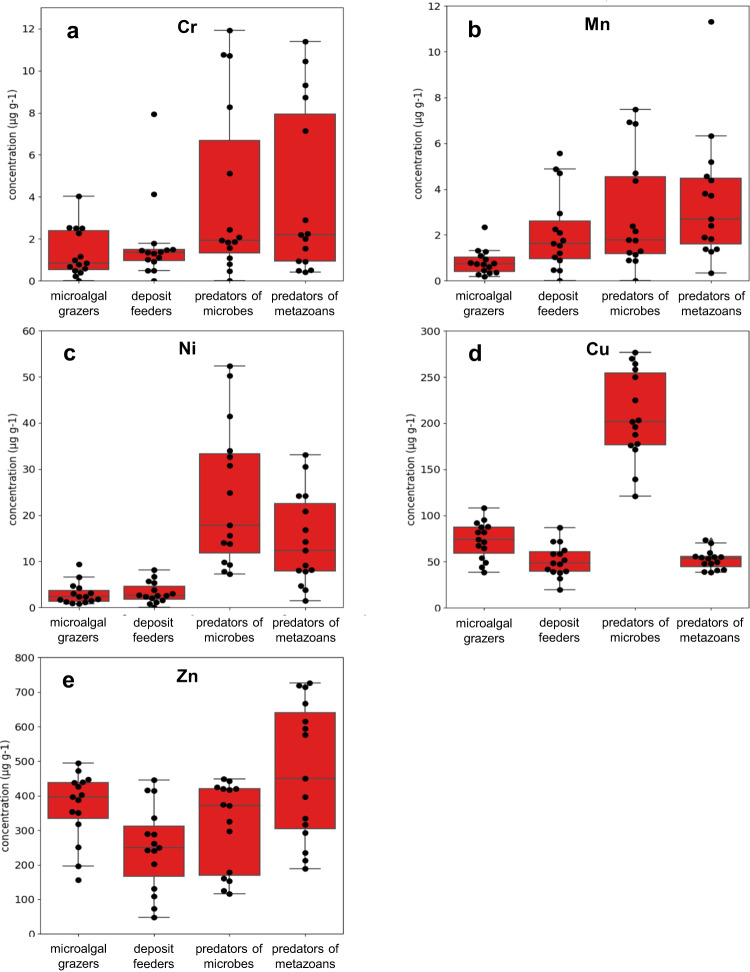
Heavy metal concentration per individual nematode biomass in the four feeding types. Selected heavy metals are: **a** Cr, **b** Mn, **c** Ni, **d** Cu and **e** Zn in the contaminated sediments of Bagnoli-Coroglio Bay. Reported are: microalgal grazers (2A), deposit feeders (1B), predators of microbes (1A) and predators of metazoans (2B). Data are represented as mean ± standard deviation (*n* = 3).

**Fig. 4:**
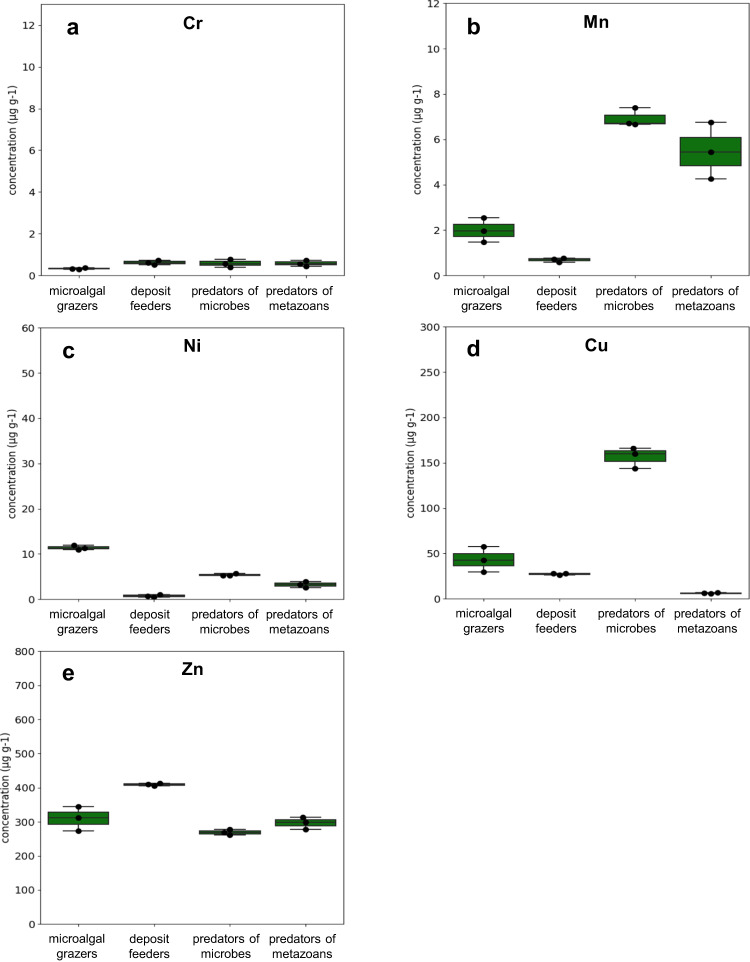
Heavy metal concentration expressed as individual nematode biomass in the four feeding types. Selected heavy metals are **a** Cr, **b** Mn, **c** Ni, **d** Cu and **e** Zn in Gabicce Mare control site. Reported are: microalgal grazers (2A), deposit feeders (1B), predators of microbes (1A) and predators of metazoans (2B). Data are represented as mean ± standard deviation (*n* = 3).

We applied the GLM also for the bioaccumulation of heavy metals calculated per individual nematode biomass (pg µg^−1^) for each feeding type estimated from the results of the X-ray microanalysis carried out on the tissues of nematodes from the contaminated and control sediments (Figs. 3a–e and 4a–e, respectively, for contaminated and control sediments). Cr and Ni bioaccumulation was significantly higher in predators of microbes (1A) and metazoans (2B) than in microalgal grazers (2A; Supplementary Table 2). Also, Cu displayed significantly higher bioaccumulation in predators of microbes (1A). Finally, Mn bioaccumulation was significantly higher in all feeding types when compared to the microalgal grazers (2A) whereas no significant differences were observed for Zn (Supplementary Table 2). The bioaccumulation of Cr in nematodes from contaminated sediments was significantly higher than in control sediments, whereas Mn and Zn did not show significant differences in nematodes from the two investigated areas (Supplementary Table 2).

**Fig. 5:**
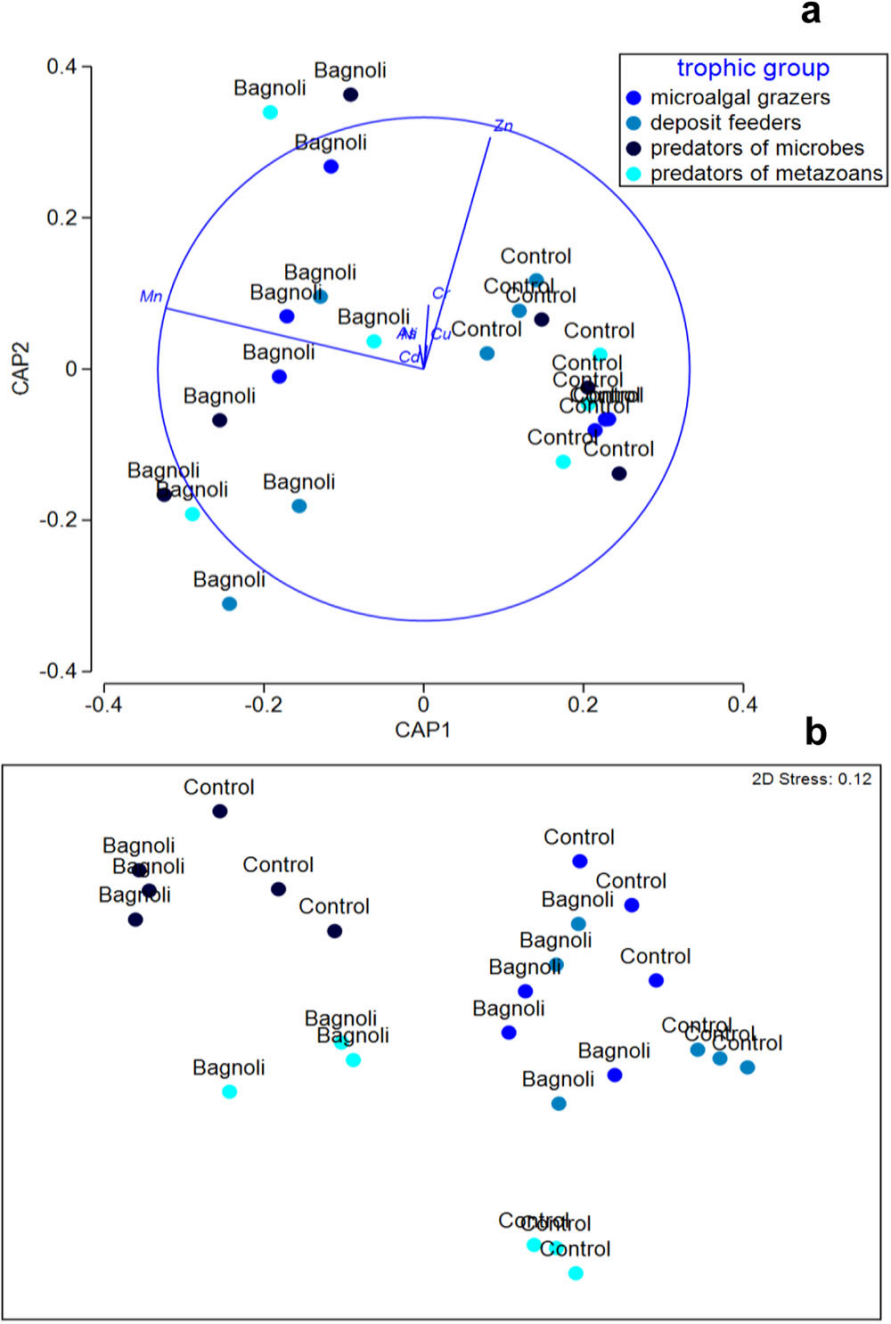
Heavy metals concentration obtained with the atomic absorption spectrophotometer analysis in the four nematode feeding types: microalgal grazers (2A), deposit feeders (1B), predators of microbes (1A) and predators of metazoans (2B). Reported are **a** the outputs of the CAP and **b** Multidimensional scaling analyses carried out in the contaminated sediments of Bagnoli-Coroglio Bay and in the Gabicce Mare control site.

Finally, we carried out multivariate analyses on the data set. The CAP analysis illustrated the segregation of nematodes from contaminated and control sediments (Fig. 5a), while the MDS analysis pointed out that predators of metazoans (2B) and of microbes (1A) in contaminated sediments segregated apart from both nematodes of control sediments and the other trophic groups of contaminated sediments (Fig. 5b).

Finally, we calculated the bioconcentration factor (BCF; Table 2). The results were always >1 for all investigated heavy metals, with highest values for Ni in predators of microbes (1A) and for As and Zn in predators of metazoans (2B).

**Table 2:** Bioconcentration factor (BCF) for the heavy metals in the four nematode feeding types in the contaminated sediments of Bagnoli-Coroglio Bay.

	As		Cd		Cr		Cu		Ni		Zn	
	avg	sd	avg	sd	Avg	sd	avg	sd	avg	sd	avg	sd
1A: predators of microbes	3.6	0.1	25.7	3.3	4.4	0.7	30.2	3.2	42.2	4.0	1.9	0.3
1B: deposit feeders	1.6	0.3	10.4	1.2	2.6	0.3	9.9	1.0	8.2	0.6	1.4	0.1
2A: microalgal grazers	1.9	0.1	23.4	5.3	2.5	0.5	10.5	2.9	6.1	0.5	1.9	0.2
2B: predators of metazoans	3.9	0.5	14.7	2.2	4.1	1.1	7.8	0.8	22.1	3.8	2.4	0.2

The bioconcentration factor is reported as mean (avg) ±standard deviation (sd).

### Metals composition in different parts of the nematode body

The results of the X-ray microanalysis of the heavy metals in different body parts of nematodes revealed that the relative contribution of Cu was higher in the head and middle body than in the tail of the deposit feeders (1B), while Zn was significantly higher in the head and middle body than tail, independently of the trophic groups in contaminated sediments (Supplementary Table 3 and Supplementary Data 1 and 2). In control sediments, the relative contribution of Mn and Zn showed different patterns: middle body > head; middle body > head and tail; head and middle body > tail, respectively, regardless of the trophic group (Supplementary Table 4).

### Heavy metals concentration and nematode body size

**Fig. 6:**
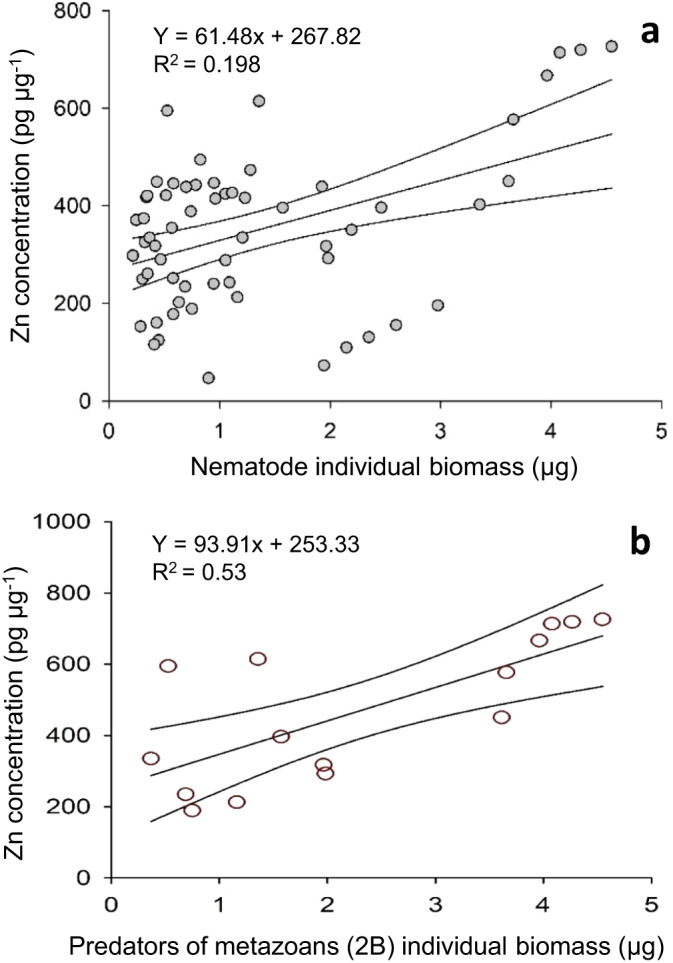
Relationship between nematode individual body size (µg) and Zn bioaccumulation. Reported are the linear regressions **a** pooling together the four nematode feeding types and **b** considering only predators of metazoans (2B).

The linear regression analysis between individual nematode body size (i.e., pooling together all individuals from all trophic groups) and heavy metal content in their tissues indicated the presence of a significant positive relationship only for Zn (Fig. 6a). Such relationship was primarily dependent upon the significant increase of Zn concentrations with the increase of the body size of the predators of metazoans (2B; Fig. 6b).

## Discussion

Xenobiotics can exert major negative effects on marine biota, which depend upon the duration of exposure and concentration^19^. In large marine organisms, the concentration of heavy metals typically increases with their size/age (bioaccumulation process) and in higher trophic levels (biomagnification processes), thus it is expected that large, long-living organisms and top predators are the components most impacted by heavy metals^20–22^. Due to their short life span and small body size, nematodes are not expected to experience significant bioaccumulation or biomagnification of heavy metals^14^ and either soil and marine nematodes can tolerate high concentrations of heavy metals (i.e., Cu, Cd, Zn, and Pb released by anthropogenic activities) in the sediments where they live^23–25^. Our results provide evidence of the bioaccumulation of heavy metals in marine nematodes. In fact, nematodes living in contaminated sediments contain in their tissue concentrations of As, Cd, and Cr significantly higher than those living in control sediments.

Our estimates of heavy metal concentrations per individual biomass obtained from the X-ray microanalysis confirm that nematodes collected in the highly contaminated sediments display higher bioaccumulation of some heavy metals (Cr and Cu) compared to those from control site. This is also evident from the multivariate analyses carried out on the bioaccumulation of all heavy metals analysed, which clearly segregated the four nematode trophic groups of contaminated sediments from those of the control site (Fig. 5).

To assess the degree of “enrichment” of each heavy metal in the nematodes compared to its concentration in the sediments, we estimated the bioconcentration factor (based on the results of the atomic absorption spectrophotometry), although it should be used with caution as the values are inversely related to the metal concentration in the environment^26^. In our study, the bioconcentration factor changed among different trophic groups suggesting different responses of the nematode feeding types to the contaminants (see below). In particular, the bioconcentration factor for As, Cr and Ni was always significantly higher in the predators of metazoans (2B) and of microbes (1A) than in microalgal grazers (2A) and deposit feeders (1B), allowing us to hypothesize a transfer of these heavy metals from the preys (microbes and tiny metazoans) to the predatory nematodes. Different accumulation mechanisms of heavy metals can be hypothesized, including (a) passive diffusion through the body surfaces, (b) adsorption on the nematode cuticle; (c) direct ingestion of heavy metals adsorbed on organic particles or contained in preys^17,23,24,27^. Heavy metals accumulation in organism’s tissues may depend also on the metal bioavailability, which in the sediment is largely controlled by their repartition in different geochemical phases^28–30^. In this regard, previous studies carried out in contaminated sediments of Bagnoli-Coroglio Bay revealed that ca 6, 15, and 40% of the total concentrations of As, Cd and Mn, respectively, are present in the most dynamic and potentially bioavailable (exchangeable/carbonate) fraction for benthic organisms^31^.

The results obtained from the X-ray microanalysis also indicate that the relative heavy metal content is not homogeneous in all parts of the nematode body and that, among the investigated metals, Cu and Zn are generally higher in the middle part of the body (oesophagus) and in the head and lower in the posterior end/tail. These findings suggest that the accumulation of heavy metals in marine nematodes does not occur through the passive diffusion or adsorption on the nematode cuticle, rather it is due to the ingestion of food items. This conclusion is consistent with previous observations on the cuticle of a specific group of soil nematodes (*Caenorhabditis elegans*), which has been reported to have a limited adsorption capacity of chemical contaminants^32^. As reported for soil nematodes we hypothesize that also marine nematodes have a limited adsorption capacity of the heavy metals through the cuticle.

In the present study, the concentrations of Cr, Cu, Mn, and Ni in the tissues of nematodes were independent from their individual body size in all feeding types. Metal accumulation depends on the balance between uptake and excretion so that our findings suggest that marine nematodes, independently from their feeding type, can be able to efficiently regulate the tissue levels of such metals during growth. At the same time, we found that Zn concentrations in nematodes were significantly and positively related to their individual biomass and such relationship was driven by predators of metazoans. Zn is an essential metal for organisms being involved in several key enzymatic reactions, but an excess amount of this metal can produce detrimental cellular and tissue effects^33,34^. Thus, our results suggest that larger size of predators of metazoans may be more vulnerable to Zn accumulation when compared to smaller counterparts and all the other trophic groups of nematodes.

### Biomagnification of heavy metals through the nematode food web

Our study based on two independent approaches and multiple heavy metals analysis revealed that nematodes belonging to different trophic groups displayed significantly different concentrations of contaminants. Among the four trophic groups investigated, predators of other metazoans and microbes displayed the highest heavy metal concentrations when compared to microalgal grazers and deposit feeders. Biomagnification of As, Cr, Mn, and Ni was observed in predators of microbes and of other metazoans, while the biomagnification of Zn was more evident in predators of metazoans. The different levels of heavy metals in predators, and the lowest concentrations in microalgal grazers and deposit feeders suggest that nematodes can accumulate these contaminants differently because of their different diet and feeding behaviours. These findings provide evidence that these organisms, at the top of the benthic microbial food web, can magnify the contaminants through the food web (from contaminated sediments to microbial components and to nematodes). Previous studies reported that benthic diatoms can accumulate low amounts of heavy metals when compared to concentrations in the sediments^35^ and this can be responsible of the lower bioaccumulation of metals observed in nematode microalgal grazers. The high contamination of the nematodes preying upon microbes could be due to their specific feeding habit. In fact, nematodes preying upon microbes can produce mucus to entrap their prey^2,36^. The mucus is known to be able to bind metals and when ingested along with microbes, increases the accumulation of heavy metals in the tissues of these nematodes^27,37–39^. The specific link between feeding mode and contamination is supported also by the comparison with deposit feeders, which generally showed the lowest heavy metal concentrations in their body as in microalgal grazers, possibly due to a diet based on organic detritus in which microbes provide a negligible energetic contribution^40^. Direct observations indicate that deposit feeders ingest organic detritus at high rates as they show a relatively constant pumping activity of the oesophagus^41^. As the passage through the nematode gut is very fast, it is plausible that not all ingested food particles are digested^41^. This allows us to hypothesize that the incorporation of heavy metals adsorbed on organic detritus leads to a lower accumulation rate than that based on the ingestion of biomass (either metazoans and microbes). However, given the complexity of the trophic pathways, specific laboratory experiments are needed to better understand the mechanisms of heavy metals incorporation in nematodes.

### Ecological implications

Nematodes play a key ecological role in all marine benthic habitats and at all depths. They are the numerically dominant component of the benthic metazoan fauna (often dominating also in terms of biomass), they feed on organic detritus, prokaryotes, protozoans, and other metazoans (as well as their carcasses) and thus represent a crucial food source for macrofauna and juvenile megafauna (including bentho-nekton components)^15^. Our results indicate that during their life, although some nematodes can be omnivorous or change their food targets, predators of metazoans (2B) and of microbes (1A) can bioaccumulate heavy metals in their body tissues more than microalgal grazers (2A) and deposit feeders (1B), resulting in potential biological effects in contaminated areas. These findings provide new insights to explain the varying tolerance of different nematode trophic groups to a mixture of heavy metals^17^ and suggest that nematodes can promote the channelling of different xenobiotics to higher trophic levels. This process has been completely overlooked so far but given the quantitative relevance of nematodes in all benthic food webs, we conclude that their contribution to xenobiotics’ bioaccumulation and biomagnification potential through the marine food webs should not be further neglected.

## Material and methods

### Study area

The Bagnoli-Coroglio Bay (Gulf of Naples, Tyrrhenian Sea) is a chronically contaminated area of the Mediterranean Sea. Here, the industrial activity, due to a steel plant using fossil coal, iron, and limestone, started in 1905 and ended in the ‘90 s. High concentrations of different metals (As, Cr, Cu, Hg, Mn, Ni, Pb, and Zn) can be found from the surface down to >2 m depth within the sediments making this area among the most polluted of the Mediterranean Sea^42–46^. In addition to the heavy metals derived from industrial activity (Cd, Cu, Hg, Pb, and Zn), the site is exposed to a natural volcanic contamination of As, Cr, Ni, and V (geogenic forcing^47^). Sediment texture is characterized by coarse sand and sandy silt at shallower depths, and fine sands, silty and silty-clay sediments in the central part of the bay^48^. Heavy metals in the sediments can be also classified in term of their potential bioavailability for the nematodes as they are present in soluble forms or associated with Fe-Mn oxides, organic matter, sulphides, and carbonates^30,31^.

### Sampling strategy

Sediment samples were collected using a Van Veen grab (25 L) in five stations: three stations located at depths ranging from 6 to 17-m, which were close to the industrial plant and characterized by the highest heavy-metal contamination, and two stations located at 8 and 14 m, respectively, in the adjacent areas characterized by a lower heavy metals contamination^46^ (Supplementary Table 1). At each station, three independent replicates of the top 1-cm of sediment were collected to analyse the level of heavy metals contamination and to extract nematodes for the determination of their individual biomass, feeding guilds, and heavy metals content in their tissues. Additional replicate sediment samples were collected in a station located in a coastal area of the Adriatic Sea (Gabicce Mare, close to the natural park Monte San Bartolo) and this was used as control.

### Heavy metals in the sediments

The concentration of As, Cd, Cu, Mn, Ni and Zn in the sediment was determined after microwave assisted acid digestion with a mixture of HNO_3,_ HF and H_2_O_2_ (EPA method 3052) and was analysed by inductively coupled plasma mass spectrometry (ICP-MS^45^.

### Nematode trophic composition and biomass

Each sediment sample was treated with ultrasounds (for 1 min 3 times, with 30 s intervals) to detach organisms from the sediment particles. This treatment has proven not to cause any damage to the nematode cuticle^49^. Then, the sediment was screened through a 0.5 mm mesh net to remove larger organisms and filtered onto a 0.02 mm mesh net to also retain the smallest nematodes^49^. The fraction retained by the 0.02 mm mesh net was re-suspended and centrifuged three times with Ludox HS40 diluted with water to a final density of 1.18 g cm^-3^ ^49^.

The unit of sampling size was adequate for subsequent nematode analyses as their abundance was in the order of ca 200–1000 ind 10 cm^−2^ (Supplementary Table 5). More than 100 specimens (on average 144–193 specimens in Bagnoli-Coroglio Bay and Gabicce Mare control site) were randomly picked up from each station and individually mounted on a temporary slide with glycerol for the identification to the species level or morphotype^50–53^ and the NeMys database^54^. The trophic group of nematodes was determined according to standard methodology^55^, which was slightly modified. Overall, 77 and 25 taxa were identified in contaminated sediments and in control site, respectively (Supplementary Tables 5 and 6). Each nematode genus/species was assigned to one of the following four trophic groups, based on the buccal morphology: (1A) predators of microbes, equipped with a small buccal cavity; (1B) deposit feeders, with a large and unarmed buccal cavity in ingest the sediments; (2A) microalgal grazers, equipped with a buccal cavity with scraping tooth or teeth and (2B) predators of metazoans / scavengers, with a buccal cavity with large jaws and able to hunt other meiofaunal organisms or to feed on their carcasses (hereafter defined as predators of metazoans).

Finally, for each specimen, biomass was calculated from their biovolume, using the Andrassy^56^ formula (V = L × W^2^ × 0.063 × 10^−5^, in which body length, L, and width, W, are expressed in µm). Each body volume was multiplied by an average density of 1.13 g cm^−3^ to obtain the wet weight, which once converted into dry weight (µg dry weight: µg wet weight = 0.25^57^), was converted into biomass assuming a carbon content of 40% of the dry weight^57^.

### Determination of heavy metals in the nematode tissues

To assess the contamination in the nematodes of different trophic groups, we used two independent approaches: (1) the determination of the heavy metal concentrations based on the atomic absorption spectrophotometer analysis and (2) the analysis of the heavy metals composition using the quantitative X-ray microanalysis. This latter analysis was carried out to provide heavy metals composition in nematodes at individual level and in different body part of the animals, since current methodologies (atomic absorption spectrophotometer and inductively coupled plasma mass spectrometry analyses) do not allow the detection of heavy metal content in single specimens characterized by very low size.

### Atomic absorption spectrophotometer analysis

All analyses were carried out in three replicates for each of the four trophic groups (i.e., pooling together specimens belonging to different species/genera of the same trophic group; Supplementary Table 6) from all the five stations of the Bagnoli-Coroglio Bay and from the Gabicce Mare control site. From each sampling station, approximately 100–150 nematodes were pooled together and analyzed for the determination of the heavy metals content, as described below. For the As, Cd, Cr, Cu, Mn, Ni and Zn concentrations, nematodes belonging to different trophic groups collected either in the Bagnoli-Coroglio Bay and in Gabicce Mare site, were digested in ultrapure HNO_3_ (2:1000, pH~2) solution. Samples were then analysed using an Agilent DUO 240FS atomic absorption spectrometer (Agilent, Santa Clara, CA 95051, USA) equipped with graphite furnace (GTA120 Graphite Tube Atomizer) and with Zeeman-effect background corrector. Argon (99.999% purity) was used as the carrier gas. Multi-element hollow cathode lamps were used as a light source. As, Cd, Cr, Cu, Mn, Ni and Zn were measured at wavelengths of 193.7, 228.8, 357.9, 324.8, 279.5, 232.0, and 213.9 nm, respectively. To improve the analytical measurements a 0.2% Pd matrix modifier in citric acid was used. Procedural blanks accounted for less than 1% of the total element concentrations in samples. The quantification of As, Cd, Cr, Cu, Mn, Ni and Zn was carried out using the calibration curve method (standard solutions of Titrisol grades from Merck) with at least three standard additions (and three measurements for each addition). The mean value obtained from each sampling area was validated with the method detection limits (LODs), calculated for each metal according to ICH Q2B: 0.08, 0.02, 0.15, 0.28, 0.06, 0.70, and 0.67 µg L^−1^, respectively, for As, Cd, Cr, Cu, Mn, Ni, and Zn^58^. To assess the accuracy of the data obtained from the instrumental analyses As, Cd, Cr, Cu, Mn, Ni and Zn were determined using BCR-414 certified reference material for plankton samples^59^. No statistically significant differences (*P* > 0.05) between certified and measured values were detected. Concentrations were reported as µg g^−1^.

### Quantitative X-ray microanalysis

The X-ray microanalysis presents two major advantages for the determination of the elemental composition of an organism: (i) higher resolution (including single cells and subcellular components) and (ii) simultaneous identification of a wide range of elements. This approach has been already tested on meiofaunal organisms belonging to the taxon of Loricifera (length 400 µm and wide 90 µm) approximately of the similar size of the nematodes investigated in the present study^60^. Such an analysis was performed on 12 individuals per each trophic group per station, using a SEM PHILIPS XL20 coupled with an EDS EDAX PHOENIX microanalysis including an ECON IV detector. X-ray microanalysis is based on X Bruker Quantax 200-Z10 with SDD detector with no liquid nitrogen. The X-ray characteristics (keV) at the minimum accelerating voltage of 15 kV are: 5.411 for Cr, 8.040 for Cu, 6.398 for Fe, 5.894 for Mn, 7.471 for Ni and 8.630 for Zn. Nematodes, belonging to different genera/species divided by trophic group, were gradually dehydrated using different concentrations of ethanol, from 70% to 80%, 90%, 95% and 100% for one night. After critical point drying, nematodes samples were graphite-coated for the X-ray microanalysis. Using an analysis frame of 13 × 13 μm and a tension of 20kv, individuals belonging to each trophic group were analysed. Replicated analyses (*n* = 3) were conducted on (i) head, (ii) middle part of body and (iii) tail of each nematode genus/species to test potential differences in heavy metals composition among different tissues/organs/parts of the body. Different heavy metals, including essential (Cu, Fe, Zn, Ni and Mn) and nonessential/borderline (Cr) elements^26^ were identified. The content of each heavy metal was expressed as a percentage of their total content in the nematode body (being the sum of all heavy metals identified by X-ray microanalysis equals to 100%). Results of the X-ray microanalysis carried out on single nematode belonging to the four trophic groups were converted into heavy metal concentrations (Cr, Cu, Mn, Ni and Zn) based on the output of the atomic absorption spectrophotometer analysis to estimate metal content per individual biomass (pg µg^-1^).

### Bioconcentration factor

The bioconcentration factor (BCF) is used to evaluate the degree of enrichment of a heavy metal in an organism compared to that in its habitat^61^. Here, we used the following equation:

$${{{{{\rm{BCF}}}}}}={{{{{{\rm{C}}}}}}}_{{{{{{\rm{nematodes}}}}}}}/{{{{{{\rm{C}}}}}}}_{{{{{{\rm{sediments}}}}}}}\,$$

where C_nematodes_ is the heavy metal concentration determined through atomic absorption spectrophotometer analysis in the nematode tissues for each trophic guild and C_sediments_ is the heavy metal concentration in the sediments. The concentration of heavy metals in nematodes and sediments was expressed as µg g^-1^.

### Statistics and reproducibility

A generalized linear model was used to describe the expected concentration of heavy metals in different nematode trophic groups from Bagnoli-Coroglio Bay and the control site and this model was fitted using maximum likelihood. This expresses the metal concentrations Y as

$${{{{{\rm{Y}}}}}}\sim {{{{{\rm{Gamma}}}}}}({{{{{\rm{\mu }}}}}},{{{{{\rm{\phi }}}}}})$$

where μ and ϕ represent the mean and dispersion parameter of the gamma distribution, respectively, and log(μ) is modelled as a linear function of the effects. Here,

$$\log ({{{{{\rm{\mu }}}}}})={{{{{{\rm{\beta }}}}}}}_{0}+{{{{{{\rm{\beta }}}}}}}_{1}{{{{{\rm{Area}}}}}}+{{{{{{\rm{\beta }}}}}}}_{2}{{{{{\rm{Trophic}}}}}}\,{{{{{\rm{Group}}}}}}$$

where β_0_ represents the intercept term, and β_1_ and β_2_ are the coefficients corresponding to the factors Area and Trophic Group, respectively.

Microalgal grazers were used as baseline since they feed on primary producers and are expected to bioaccumulate heavy metals to less extent compared with the other nematode feeding types^35^.

Analyses were conducted using the GLM procedure in R.

To visualize differences in heavy metals content among different trophic groups and areas, biplots after a Canonical Analysis of Principal Coordinates (CAP) were made^62^. The similarity matrix in the heavy metals content of nematodes from Bagnoli-Coroglio Bay and the Gabicce Mare control site, based on the Bray–Curtis similarity (square root transformed), was applied to produce a non-metric multidimensional scaling two-dimensional plot (MDS).

PERMANOVA was applied to test for differences in the heavy metals composition (obtained by the X-ray microanalysis) in different body parts comparing head, middle body, and tail in the four nematode trophic groups in Bagnoli-Coroglio Bay and in the Gabicce Mare control site using a three-way distance-based permutational analyses of variance based on unrestricted permutations of the raw data. All univariate analyses were carried out on Euclidean distance of untransformed data, using 999 permutations of the residuals under a reduced model. For all analyses, when significant differences were observed, pair-wise tests were also carried out. Because of the restricted number of unique permutations in the pair-wise tests, *p* values were obtained from Monte Carlo sampling method^63^.

PERMANOVA, CAP and MDS analyses were performed using the routines included in the PRIMER v7 and PERMANOVA software^63^.

Finally, a linear regression analysis was used to investigate the relationships between body size of nematodes within each trophic group and their heavy metals content.

### Reporting summary

Further information on research design is available in the Nature Portfolio Reporting Summary linked to this article.

### Supplementary information

Supplementary Information

Description of Additional Supplementary Files

Supplementary Data 1

Supplementary Data 2

Supplementary Data 3

Reporting Summary

## Data Availability

The source data behind the graphs can be found in Supplementary Data 3. All other data supporting the findings of this study can be obtained from the corresponding author on reasonable request (if appropriate).
