# Supplementary material for: Bioaccumulation and biomagnification of heavy metals in marine micro-predators
Source: Commun Biol. 2023 Nov 27;6:1206. doi: 10.1038/s42003-023-05539-x (PMC10682414; doi:10.1038/s42003-023-05539-x)
Supplement: Supplementary file 1 — Supplementary Information [file 42003_2023_5539_MOESM1_ESM.pdf]

Supplementary information

Bioaccumulation and biomagnification of heavy metals in marine micro-predators

Roberto Danovaro[1,2*@], Adele Cocozza di Montanara[1,3*], Cinzia Corinaldesi[4*], Antonio Dell'Anno[1*], Silvia Illuminati[1], Trevor J. Willis[5], Cristina Gambi[1*]

[1]Dipartimento di Scienze della Vita e dell'Ambiente, Università Politecnica delle Marche, Via Brecce Bianche, 60131 Ancona, Italy.

[2] Nature Biodiversity Future Centre, Italy.

[3]Dipartmento di Scienze e Tecnologie, Università degli Studi di Napoli Parthenope, Centro Direzionale, 80143 Napoli, Italy.

[4]Dipartimento di Scienze e Ingegneria della Materia, dell'Ambiente ed Urbanistica, Università Politecnica delle Marche, Via Brecce Bianche, 60131 Ancona, Italy.

[5]Fano Marine Centre, Stazione Zoologica Anton Dohrn, 61032 Fano, Italy.

* *These authors contributed equally*

[@]Corresponding author: r.danovaro@univpm.it

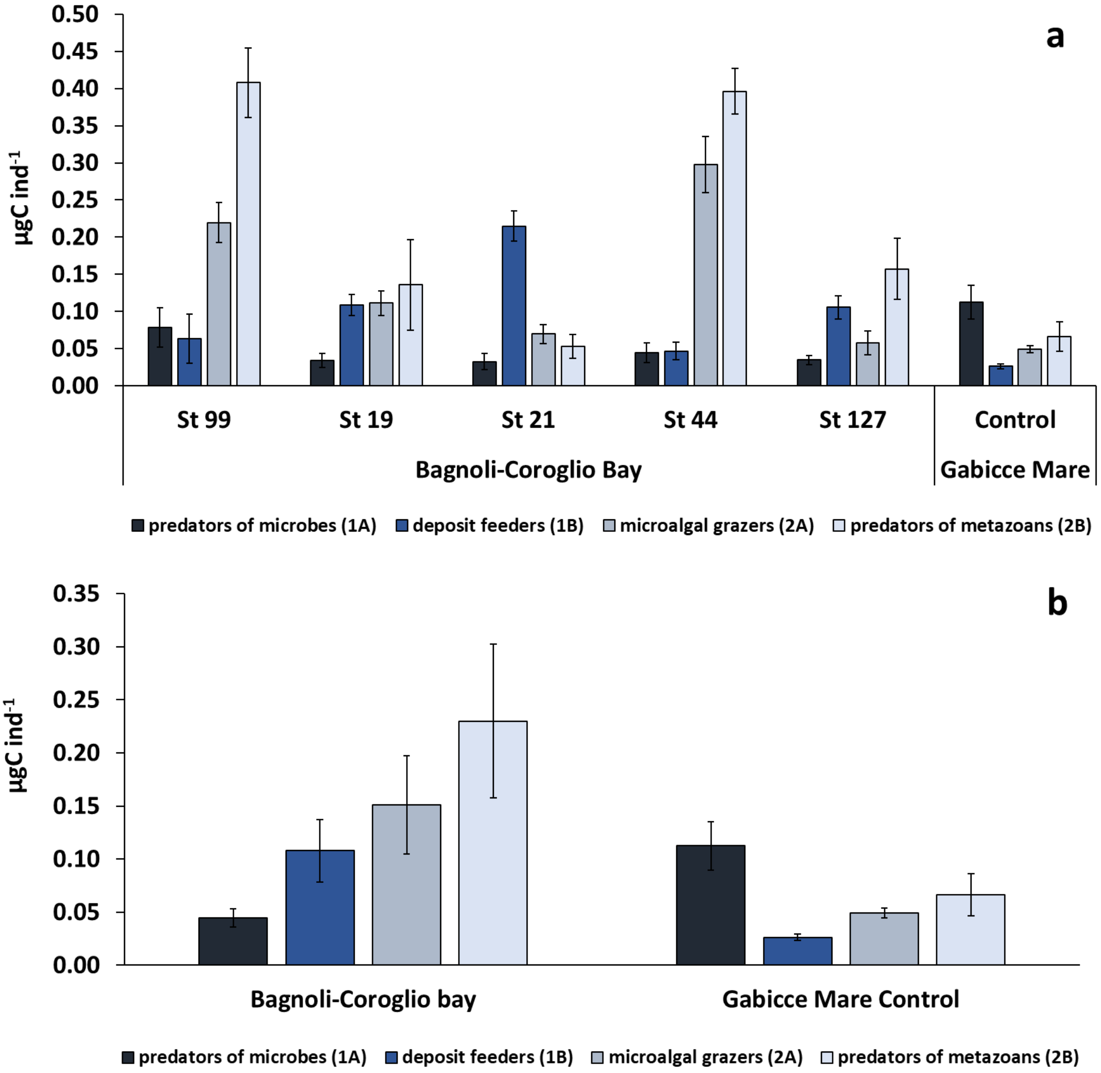

Supplementary Figure 1. Individual nematode biomass for each trophic group in samples collected in the contaminated sediments of Bagnoli-Coroglio Bay (Tyrrhenian Sea) and in the control site located in the Gabicce Mare (Adriatic Sea). Reported are a) average value and standard deviation for each trophic group in all stations and b) average values and standard errors for each trophic group in the contaminated and control sediments.
Data are represented as mean ± standard deviation (n=3).

Supplementary Table 1. Spatial distribution of the concentration of As, Cd, Cr, Cu, Ni, Mn, and Zn in contaminated sediments of the Bagnoli-Coroglio Bay and in the control site of Gabicce Mare (Adriatic Sea). Concentrations are reported as mean ± standard deviation (sd). Latitude (Lat) and Longitude (Long) are also reported.

Area	Station	Lat.	Long.	As		Cd		Cr		Cu		Ni		Mn		Zn	
		N	E	μg g^{-1}	sd	μg g^{-1}	sd	μg g^{-1}	sd	μg g^{-1}	sd	μg g^{-1}	sd	μg g^{-1}	sd	μg g^{-1}	sd
Bagnoli-Coroglio	99	40.817	14.135	31.70	2.54	0.35	0.04	58.35	5.25	8.00	1.04	11.00	1.21	1195	95.60	115	14.95
	19	41.353	14.272	104.00	11.44	1.92	0.17	35.81	4.30	35.30	3.53	13.60	1.77	1472	132.48	745	74.50
	21	41.349	14.259	64.80	5.83	1.09	0.14	15.70	1.73	28.30	2.55	8.00	0.96	698	69.80	431	34.48
	44	41.347	14.277	48.10	5.77	1.54	0.18	31.80	3.18	30.60	3.98	15.70	2.04	1800	144.00	740	96.20
	127	40.793	14.180	21.70	1.74	0.39	0.04	12.70	1.02	15.30	1.68	5.20	0.47	874	113.62	149	13.41
Gabicce Mare	control	43.966	12.764	6.80	0.75	0.15	0.01	22.53	2.48	8.90	1.16	16.50	1.32	338	40.56	56	4.50

Supplementary Table 2. GLM (Log-Gamma Model) estimates of differences in metal concentrations among nematode trophic groups and areas (control site of Gabicce Mare vs contaminated sediments of Bagnoli-Coroglio Bay), expressed as odds ratios. 95% confidence limits are asymmetric around the ratio estimate because error estimates are calculated on the log scale and are therefore multiplicative on the arithmetic scale.

	Contrast	Effect ratio (relative to predators of microalgae)	Lower 95% confidence limit for ratio	Upper 95% confidence limit for ratio	t-value	P
Cr	Deposit feeders	1.42	0.78	2.61	1.14	0.2584
	Predators of microbes	2.81	1.53	5.15	3.33	0.0014
	Predators of metazoans	2.82	1.54	5.18	3.35	0.0014
	Area (control : contaminated sediments)	0.22	0.12	0.38	-5.20	<0.0001
Mn	Deposit feeders	2.06	1.25	3.39	2.85	0.0057
	Predators of microbes	3.58	2.18	5.88	5.03	<0.0001
	Predators of metazoans	3.96	2.42	6.51	5.43	<0.0001
	Area (control : contaminated sediments)	1.59	0.99	2.56	1.95	0.0553
Ni	Deposit feeders	0.58	0.33	0.99	-1.98	0.0519
	Predators of microbes	4.27	2.47	7.39	5.20	<0.0001
	Predators of metazoans	2.59	1.50	4.49	3.41	0.0011
	Area (control : contaminated sediments)	0.76	0.45	1.28	-1.02	0.3129
Cu	Deposit feeders	0.69	0.56	0.85	3.51	0.0008
	Predators of microbes	3.00	2.43	3.70	1.02	<0.0001
	Predators of metazoans	0.60	0.49	0.74	4.75	<0.0001
	Area	0.48	0.39	0.58	-7.24	<0.0001

	(control : contaminated sediments)					
Zn	Deposit feeders	0.77	0.59	1.00	-1.95	0.0549
	Predators of microbes	0.85	0.66	1.10	-1.24	0.219
	Predators of metazoans	1.23	0.95	1.59	1.54	0.1273
	Area<br>(control : contaminated sediments)	0.97	0.76	1.24	-0.24	0.8084

Supplementary Table 3. Output of the PERMANOVA analysis carried out to test for differences in heavy metal composition and accumulation in different body parts (head, middle body, and tail) of the four nematodes trophic groups in the five stations of the contaminated sediments of Bagnoli-Coroglio Bay (DF=degrees of freedom; MS=mean square; F=F statistic; P=probability level; ***=p<0.001; **=P<0.01; *=P<0.05; ns: not significant).

	Source	df	MS	F	P
All heavy metals	Station (St)	4	3919.4	9.750	***
	Trophic group (TG)	3	4103.9	10.209	***
	Body part (BP)	2	1654.4	4.116	**
	St x TG	12	2486.1	6.185	***
	St x BP	8	122.24	0.304	ns
	Tg x BP	6	781.4	1.944	*
	St x TG x BP	24	179.14	0.446	ns
	Residuals	120	401.98		
	total	179			
Cr	Station (St)	4	40.571	4.5655	**
	Trophic group (TG)	3	19.697	2.2166	ns
	Body part (BP)	2	6.5437	0.73637	ns
	St x TG	12	23.222	2.6132	**
	St x BP	8	3.6075	0.40596	ns
	Tg x BP	6	0.82124	9.24E-02	ns
	St x TG x BP	24	4.9029	0.55173	ns
	Residuals	120	8.8864		
	total	179			
Cu	Station (St)	4	265.28	7.5055	**
	Trophic group (TG)	3	888.57	25.14	**
	Body part (BP)	2	5.1917	0.14689	ns
	St x TG	12	217.27	6.1472	**
	St x BP	8	15.286	0.43248	ns
	Tg x BP	6	88.964	2.5171	*
	St x TG x BP	24	22.942	0.64911	ns
	Residuals	120	35.344		
	total	179			
Fe	Station (St)	4	929.2	6.9957	***
	Trophic group (TG)	3	2713.5	20.429	***
	Body part (BP)	2	756.65	5.6966	**
	St x TG	12	780.57	5.8768	***
	St x BP	8	41.248	0.31055	ns
	Tg x BP	6	416.02	3.1322	*

	St x TG x BP	24	68.437	0.51525	ns
	Residuals	120	132.82		
	total	179			
Mn	Station (St)	4	17.055	2.5904	*
	Trophic group (Tg)	3	7.0098	1.0647	ns
	Body part (Bp)	2	10.765	1.635	ns
	St x TG	12	20.444	3.1051	**
	St x BP	8	3.1804	0.48304	ns
	Tg x BP	6	2.712	0.4119	ns
	St x TG x BP	24	4.2578	0.64667	ns
	Residuals	120	6.5842		
	total	179			
Ni	Station (St)	4	60.458	4.2744	**
	Trophic group (TG)	3	58.697	4.1499	**
	Body part (BP)	2	6.7844	0.47966	ns
	St x TG	12	50.654	3.5813	***
	St x BP	8	5.0966	0.36034	ns
	Tg x BP	6	12.056	0.85235	ns
	St x TG x BP	24	11.905	0.84168	ns
	Residuals	120	14.144		
	total	179			
Zn	Station (St)	4	2606.9	12.766	**
	Trophic group (TG)	3	416.48	2.0396	ns
	Body part (BP)	2	868.5	4.2531	*
	St x TG	12	1394	6.8264	**
	St x BP	8	53.818	0.26355	ns
	Tg x BP	6	260.82	1.2773	ns
	St x TG x BP	24	66.698	0.32663	ns
	Residuals	120	204.2		
	total	179			

Supplementary Table 4. Output of the PERMANOVA analysis carried out to test for differences in heavy metal composition and accumulation in different body parts (BP) (head (H), middle body (B), and tail (T)) of the four trophic groups (TG): 1A (predators of microbes); 1B (deposit feeders); 2A (microalgal grazers) and 2B (predators of metazoans) in the Gabicce Mare control site (DF=degrees of freedom; MS=mean square; F=F statistic; P=probability level; ***=p<0.001; **=P<0.01; *=P<0.05; ns: not significant).

	Source	df	MS	F	P	Head	Body	Tail	1A	1B	2A	2B	Body part	Trophic Group
All heavy metals	Trophic group (TG)	3	1005.4	2.50	*									2B≠1A
	Body part (BP)	2	1924.5	4.79	**								H&B≠T	
	TG x BP	6	236.1	0.59	ns									
	Residuals	24	401.95											
	Total	35												
Cr	Trophic group (TG)	3	5.3	2.27	ns									
	Body part (BP)	2	4.8	2.07	ns									
	TG x BP	6	1.4	0.58	ns									
	Residuals	24	2.3											
	Total	35												
Cu	Trophic group (TG)	3	369.7	3.11	*									2B>1A
	Body part (BP)	2	14.2	0.12	ns									
	TG x BP	6	37.2	0.31	ns									
	Residuals	24	119.0											
	Total	35												
Fe	Trophic group (TG)	3	29.3	0.32	ns									
	Body part (BP)	2	890.7	9.74	***								B>H&T	
	TG x BP	6	69.9	0.76	ns									

	Residuals	24	91.4				
	Total	35					
Mn	Trophic group (TG)	3	5.4	0.82	ns		
	Body part (BP)	2	23.6	3.62	*	B>H	
	TG x BP	6	8.3	1.28	ns		
	Residuals	24	6.5				
	Total	35					
Ni	Trophic group (TG)	3	2.9	0.34	ns		
	Body part (BP)	2	14.8	1.72	ns		
	TG x BP	6	13.0	1.51	ns		
	Residuals	24	8.6				
	Total	35					
Zn	Trophic group (TG)	3	592.8	3.41	*		2B>1A
	Body part (BP)	2	976.5	5.61	***	H&B>T	
	TG x BP	6	106.4	0.61	ns		
	Residuals	24	174.1				
	Total	35					

Supplementary Table 5. Nematode abundance and species richness in the top 1 cm of sediment samples collected in the contaminated sediments of Bagnoli-Coroglio Bay and Gabicce Mare control site.

	Bagnoli Coroglio Bay					Gabicce Mare
Nematode	St99	St19	St21	St44	St127	control
Average abundance (ind 10 cm^{-2})	268	326	193	1013	715	364
Species Richness	24	24	27	23	23	25

Supplementary Table 6. Nematode morphospecies, feeding type and relative abundance in the top 1 cm of samples collected in the contaminated sediments of Bagnoli-Coroglio Bay and in the control site of Gabicce Mare.

Morphospecies	Feeding type	Bagnoli-Coroglio Bay					Gabicce Mare
		St19	St99	St21	St44	St127	Control
Adoncholaimus sp1	Predator of metazoans (2B)				1		3
Adoncholaimus sp3	Predator of metazoans (2B)	4	1	14	18	6	
Aegialoalaimus sp2	Predator of microbes (1A)					17	
Amphimonhystrella sp1	Deposit feeder (1B)				3		
Anticoma sp1	Predator of microbes (1A)	11					
Anticyathus sp1	Deposit feeder (1B)			2			
Antomicron sp1	Predator of microbes (1A)			1			
Axonolaimus p3	Deposit feeder (1B)					2	
Axonolaimus sp1	Deposit feeder (1B)	15		1	4		26
Bathyeurystomina sp1	Predator of metazoans (2B)	1					1
Bathyeurystomina sp4	Predator of metazoans (2B)			4			
Bathyeurystomina sp6	Predator of metazoans (2B)			1			
Bolbolaimus sp1	Predator of metazoans (2B)			1			
Camacolaimus sp1	Microalgal grazer (2A)			1			
Cephalanticoma sp1	Microalgal grazer (2A)	14					31
Ceramonema sp1	Predator of microbes (1A)		1				
Chaetonema sp1	Deposit feeder (1B)				55		
Chromadorita sp1	Microalgal grazer (2A)			2	1		
Coninckia sp1	Predator of microbes (1A)			1			
Crenopharynx sp1	Microalgal grazer (2A)						4
Cyartonema sp1	Predator of microbes (1A)					1	5
Daptonema sp1	Deposit feeder (1B)	12			4	4	22
Desmodora sp1	Microalgal grazer (2A)	1					
Desmodora sp10	Microalgal grazer (2A)	6	14	8			
Desmodora sp12	Microalgal grazer (2A)		3				
Desmodora sp16	Microalgal grazer (2A)	9	6		21		
Desmodora sp17	Microalgal grazer (2A)				7	1	
Desmodora sp18	Microalgal grazer (2A)		4				
Diplopeltoides sp1	Predator of microbes (1A)			4			
Eleutherolaimus sp1	Deposit feeder (1B)		4			6	
Elzalia sp8	Deposit feeder (1B)				2		
Enoplolaimus sp1	Predator of metazoans (2B)	1		3	5		1
Epacanthion sp1	Predator of metazoans (2B)						2
Epsilonema sp1	Predator of microbes (1A)		40	1			
Euchromadora sp3	Microalgal grazer (2A)		1				
Gammanema sp1	Predator of metazoans (2B)		6				
Halalaimus sp6	Predator of microbes (1A)			5	2		

Halalaimus sp7	Predator of microbes (1A)					4	
Innocuonema sp 2	Microalgal grazer (2A)	2					32
Innocuonema sp1	Microalgal grazer (2A)		6	13			2
Latronema sp1	Predator of metazoans (2B)					2	
Longicyatholaimus sp1	Predator of microbes (1A)			1			
Marylynnia sp2	Predator of microbes (1A)				1	19	
Marylynnia sp4	Predator of microbes (1A)					4	
Metachromadora sp2	Predator of metazoans (2B)		5				
Metacyatholaimus sp1	Microalgal grazer (2A)						2
Metadesmolaimus sp1	Deposit feeder (1B)	9		2	13		
Metadesmolaimus sp2	Deposit feeder (1B)					1	
Metalinhomoeus sp1	Deposit feeder (1B)						2
Metalinhomoeus sp3	Deposit feeder (1B)						3
Metoncholaimus sp1	Predator of metazoans (2B)	1			10		15
Microlaimus sp1	Microalgal grazer (2A)	9	6		2	6	
Monhystrella sp2	Deposit feeder (1B)			3			
Odontanticoma sp1	Microalgal grazer (2A)						4
Oncholaimellus sp1	Predator of metazoans (2B)						1
Onyx sp 1	Predator of metazoans (2B)		6				
Parachromadorita sp1	Predator of microbes (1A)		1		14	2	
Paracyatholaimoides sp1	Microalgal grazer (2A)					2	
Paracyatholaimoides sp3	Microalgal grazer (2A)				6		
Paracyatholaimus sp1	Microalgal grazer (2A)			18			
Paracyatholaimus sp4	Microalgal grazer (2A)				1		
Paramesacanthion sp1	Predator of metazoans (2B)			5		7	
Paramonhystera sp1	Deposit feeder (1B)	8				1	15
Perspiria sp1	Microalgal grazer (2A)		1				
Phanodermopsis sp1	Predator of microbes (1A)	17				1	
Phanodermopsis sp2	Predator of microbes (1A)						2
Polysigma sp2	Microalgal grazer (2A)					1	
Praeacanthonchus sp1	Microalgal grazer (2A)			2			
Prochaetosoma sp1	Microalgal grazer (2A)		1				
Prochromadora sp1	Microalgal grazer (2A)					1	1
Prochromadorella sp1	Microalgal grazer (2A)	10	5	6	2		9
Prochromadorella sp3	Microalgal grazer (2A)					1	
Rhinema sp1	Microalgal grazer (2A)		1				
Rhips sp1	Microalgal grazer (2A)		2			3	
Richtersia sp10	Deposit feeder (1B)		7			1	
Richtersia sp11	Deposit feeder (1B)	1	2	38		22	
Richtersia sp12	Deposit feeder (1B)		16				
Setosabatieria sp1	Deposit feeder (1B)				1		
Spilophorella sp1	Microalgal grazer (2A)	1					1
Spirinia sp1	Microalgal grazer (2A)		4	1			

Steineridora sp1	Microalgal grazer (2A)				2			
Stylotheristus sp1	Deposit feeder (1B)							3
Syringolaimus sp1	Predator of metazoans (2B)			5				
Terschellingia sp1	Predator of microbes (1A)	3		1				4
Thalassironus sp1	Predator of metazoans (2B)			1				
Thalassoalaimus sp1	Predator of microbes (1A)							2
Tubolaimoides sp4	Predator of microbes (1A)			4		1		
