# Supplementary material for: Bioaccumulation and biomagnification of heavy metals in marine micro-predators
Source: Commun Biol. 2023 Nov 27;6:1206. doi: 10.1038/s42003-023-05539-x (PMC10682414; doi:10.1038/s42003-023-05539-x)
Supplement: Supplementary file 2 — Description of Additional Supplementary Files [file 42003_2023_5539_MOESM2_ESM.pdf]

Description of Additional Supplementary Files

File name: Supplementary Data 1

Description: Results of X-ray microanalysis in different parts of the four nematode trophic groups.

File Name: Supplementary Data 2

Description: Output of the pair-wise tests of the PERMANOVA analyses on data reported in the Supplementary Data 1.

File Name: Supplementary Data 3

Description: The source data behind the graphs 1-6.
