# Supplementary material for: Bioaccumulation and biomagnification of heavy metals in marine micro-predators
Source: Commun Biol. 2023 Nov 27;6:1206. doi: 10.1038/s42003-023-05539-x (PMC10682414; doi:10.1038/s42003-023-05539-x)
Supplement: Supplementary file 6 — Reporting Summary [file 42003_2023_5539_MOESM6_ESM.pdf]

nature portfolio

Corresponding author(s): Roberto Danovaro

Last updated by author(s): 2023/10/31

Reporting Summary

Nature Portfolio wishes to improve the reproducibility of the work that we publish. This form provides structure for consistency and transparency in reporting. For further information on Nature Portfolio policies, see our Editorial Policies and the Editorial Policy Checklist.

Statistics

For all statistical analyses, confirm that the following items are present in the figure legend, table legend, main text, or Methods section.

n/a	Confirmed	
☐	☒	The exact sample size (*n*) for each experimental group/condition, given as a discrete number and unit of measurement
☐	☒	A statement on whether measurements were taken from distinct samples or whether the same sample was measured repeatedly
☐	☒	The statistical test(s) used AND whether they are one- or two-sided<br>*Only common tests should be described solely by name; describe more complex techniques in the Methods section.*
☐	☒	A description of all covariates tested
☒	☐	A description of any assumptions or corrections, such as tests of normality and adjustment for multiple comparisons
☐	☒	A full description of the statistical parameters including central tendency (e.g. means) or other basic estimates (e.g. regression coefficient) AND variation (e.g. standard deviation) or associated estimates of uncertainty (e.g. confidence intervals)
☒	☐	For null hypothesis testing, the test statistic (e.g. *F*, *t*, *r*) with confidence intervals, effect sizes, degrees of freedom and *P* value noted<br>*Give P values as exact values whenever suitable.*
☒	☐	For Bayesian analysis, information on the choice of priors and Markov chain Monte Carlo settings
☒	☐	For hierarchical and complex designs, identification of the appropriate level for tests and full reporting of outcomes
☒	☐	Estimates of effect sizes (e.g. Cohen's *d*, Pearson's *r*), indicating how they were calculated

Our web collection on statistics for biologists contains articles on many of the points above.

Software and code

Policy information about availability of computer code

Data collection	no software was used for the data collection.
Data analysis	data elaboration supported by Excel, PRIMER & PERMANOVA, GLM procedure in R.

For manuscripts utilizing custom algorithms or software that are central to the research but not yet described in published literature, software must be made available to editors and reviewers. We strongly encourage code deposition in a community repository (e.g. GitHub). See the Nature Portfolio guidelines for submitting code & software for further information.

Data

Policy information about availability of data

All manuscripts must include a data availability statement. This statement should provide the following information, where applicable:

- Accession codes, unique identifiers, or web links for publicly available datasets
- A description of any restrictions on data availability
- For clinical datasets or third party data, please ensure that the statement adheres to our policy

All relevant data are included in the main text and in the Supplementary Information data. The datasets generated during and/or analysed during the current study are available from the corresponding author on reasonable request.

Research involving human participants, their data, or biological material

Policy information about studies with human participants or human data. See also policy information about sex, gender (identity/presentation), and sexual orientation and race, ethnicity and racism.

Reporting on sex and gender	n/a
Reporting on race, ethnicity, or other socially relevant groupings	n/a
Population characteristics	n/a
Recruitment	n/a
Ethics oversight	n/a

Note that full information on the approval of the study protocol must also be provided in the manuscript.

Field-specific reporting

Please select the one below that is the best fit for your research. If you are not sure, read the appropriate sections before making your selection.

☐ Life sciences ☐ Behavioural & social sciences ☒ Ecological, evolutionary & environmental sciences

For a reference copy of the document with all sections, see nature.com/documents/nr-reporting-summary-flat.pdf

Ecological, evolutionary & environmental sciences study design

All studies must disclose on these points even when the disclosure is negative.

Study description	Here we investigated the heavy metals accumulation (i.e., As, Cd, Cr, Cu, Fe, Mn, Ni, and Zn) in nematodes living in contaminated and control sediments using two independent approaches to test their bioaccumulation and potential contribution to biomagnification of xenobiotics. All analyses have been carried out in three replicates for each of the 6 sampling sites.
Research sample	Nematodes are tolerant to most pollution sources and are consequently reported also in highly contaminated sediments, where they are directly exposed to contaminants for their entire life cycle (given the lack of planktonic larvae). Nematodes can bioaccumulate xenobiotics, but due to their extremely short life span and tiny body size, this aspect has been so far completely neglected. More than one-hundred specimens (on average 144-193 specimens in Bagnoli-Coroglio Bay and control site) were randomly picked up from each sampling station and individually mounted on a temporary slide with glycerol for the identification to the species level or morphotype.
Sampling strategy	Sediment samples were collected using a Van Veen grab in five stations at depths ranging from 6 to 17-m in the highly contaminated sediments of the Bagnoli-Coroglio Bay. Additional sediment samples were collected in a pristine coastal area of the Adriatic Sea (Gabicce Mare, close to the natural park Monte San Bartolo) and these samples were considered as control. At each station, three independent replicates of the top 1 cm of sediment were collected to assess the content of heavy metals and to determine the nematodes individual biomass and four feeding types (predators of microbes, predators of metazoans, deposit feeders and microalgal grazers), and the accumulation of heavy metals in their tissues.
Data collection	Data were analyzed and recorded at the laboratory of Marine Ecology and Biology at the Department of Life and Environmental Sciences of the Polytechnic University of Marche, Italy.
Timing and spatial scale	Sediment samples have been collected in July 2017 in five stations at depths ranging from 6 to 17-m in the highly contaminated sediments of the Bagnoli-Coroglio Bay and in a pristine coastal area of the Adriatic Sea (Gabicce Mare, close to the natural park Monte San Bartolo). The analysis of the heavy metals in nematode tissues have been completed in Spring 2021.
Data exclusions	No data were excluded from the study.
Reproducibility	Heavy metals accumulation in the four nematode trophic groups (predators of microbes, predators of metazoans, deposit feeders and microalgal grazers) were analysed in replicates using two independent approaches: 1) the determination of the heavy metal concentrations based on the atomic absorption spectrophotometer analysis and 2) the analysis of the heavy metal composition using the quantitative X-ray microanalysis. All protocols are described in the manuscript.
Randomization	Samples were collected in stations characterized by different levels of heavy metals contamination (including a control) representing the local condition of the area.
Blinding	Blinding is no relevant for the analyses performed in this study.

Did the study involve field work? ☒ Yes ☐ No

Field work, collection and transport

Field conditions	Sediment samples were collected in July 2017 on board the R/V Vettoria with excellent sea conditions, seawater temperature on average: 25°C.
Location	Sampling stations<br>Bagnoli-Coroglio: St 99; N 40.817, E 14.135, depth: 8m; St 19; N 41.353, E 14.272, depth: 6m; St 21; N 41.349, E 14.259, depth: 17m St 44; N 41.347, E 14.277, depth: 6m; St 127; N 40.793, E 14.180, depth: 14m; Gabicce Mare: control; N 43.966, E 12.764, depth: 5 m
Access & import/export	Samples collection was carried out on board the R/V Vettoria. The study area is in front the coast of Naples. Sampling activities were carried out in the framework of a national project coordinated by the Stazione Zoologica Anton Dohrn. The activities were carried out according to the local port authorities. Sediment samples were immediately stored with dry ice on board and transported by car to Ancona at the laboratory of Marine Biology and Ecology where the laboratory analyses were performed. Samples collected in Gabicce Mare were immediately stored with dry ice and transported by car to Ancona at the laboratory of Marine Biology and Ecology for the analyses.
Disturbance	The sampling activities have been minimised to avoid any impact on the sea bottom characterised by soft sediments. The sampling sites do not occur in habitats characterised by endangered species that require special management and attention.

Reporting for specific materials, systems and methods

We require information from authors about some types of materials, experimental systems and methods used in many studies. Here, indicate whether each material, system or method listed is relevant to your study. If you are not sure if a list item applies to your research, read the appropriate section before selecting a response.

Materials & experimental systems

n/a	Involved in the study	
☒	☐	Antibodies
☒	☐	Eukaryotic cell lines
☒	☐	Palaeontology and archaeology
☐	☒	Animals and other organisms
☒	☐	Clinical data
☒	☐	Dual use research of concern
☒	☐	Plants

Methods

n/a	Involved in the study	
☒	☐	ChIP-seq
☒	☐	Flow cytometry
☒	☐	MRI-based neuroimaging

Animals and other research organisms

Policy information about studies involving animals; ARRIVE guidelines recommended for reporting animal research, and Sex and Gender in Research

Laboratory animals	This study does not involve laboratory animals.
Wild animals	This study involves free-living marine nematodes. Sediment samples have been collected using a Van Veen grab, specimens were extracted from the sediment using a density gradient of LUDOX according to standard protocols. Once extracted, nematodes were identified and prepared for the determination of heavy metals in their tissues. The unit of sampling size was adequate for subsequent nematode analyses as their abundance was in the order of ca 200-1000 ind 10 cm-2 in the sampling sites.
Reporting on sex	No sex analysis has been included in the present investigation
Field-collected samples	Nematodes were extracted from sediments stored at -20°C using a density gradient of LUDOX according to standard protocols reported in the material and Methods. Once extracted, nematodes were identified and prepared for the determination of heavy metals in their tissues. All procedures are detailed in the manuscript.
Ethics oversight	No ethical approval is requested for this research. All protocols are described in the manuscript and their references are provided in the text.

Note that full information on the approval of the study protocol must also be provided in the manuscript.

Plants

Seed stocks	n/a
Novel plant genotypes	n/a
Authentication	n/a
